# The Spectrum of Malignant Neoplasms among Liver Transplant Recipients: Sociodemographic Factors, Mortality, and Hospital Burden

**DOI:** 10.7150/ijms.66533

**Published:** 2022-01-09

**Authors:** Maryam Haider, Anusha Bapatla, Rana Ismail, Ahmed J Chaudhary, Sana Iqbal, Syed M Haider

**Affiliations:** Department of Internal Medicine, Detroit Medical Center/Wayne State University-Sinai Grace Hospital, Detroit, USA.

**Keywords:** Liver Transplantation, Transplant Recipients, Neoplasms, hepatocellular carcinoma (HCC), Lymphoma, Gastrointestinal Neoplasms.

## Abstract

**Objective:** To determine the nationwide prevalence of malignant neoplasms (excluding hepatocellular carcinoma-HCC) in hospitalized liver transplant recipients and to study the hospital utilization, and mortality to the incidence of malignancies. To the best of our knowledge, few epidemiological studies addressed outcomes in post-liver transplant patients, such as the annual number of hospitalizations, mortality, patient characteristics regarding malignancies.

**Methods:** NIS database was queried between 2016 and 2018 to retrieve records of patients admitted with a principal or secondary diagnosis of liver transplant following *the International Classification of Diseases, tenth Revision (ICD-10)*. The population was divided into case and control groups according to the presence and absence of malignant neoplasm (MN) except for HCC. We also compared the incidence of MN in LTX patients and non-LTX matched cohort.

**Results:** A total of 7.28% admissions were associated with malignant neoplasms (except HCC) in LTX patients. Lymphomas, respiratory, gastrointestinal (excluding HCC), leukemia, and head/neck were commonest cancers with estimated admission rates of 0.97%, 0.90%, 0.80%, 0.53%, and 0.49%, respectively. Lung cancer was the most frequent malignant neoplasm among White and Black racial/ethnic groups (15.78% and 14.8%), whereas lymphoma was pervasive among Hispanics (20.3%). Lung cancer had the highest in-hospital mortality (10.55%), followed by the cancer of the nervous system (9.09%). The LTX and non-LTX cohort comparison showed that LTX patients are at increased risk of head and neck cancers, skin cancers, lymphomas, tumors, and Myelodysplastic syndrome. According to a multivariate analysis, a statistically significant association existed between malignant neoplasms in LTX patients and the following factors: increasing age (P < .001), higher mortality (P < .001), females with 29% lesser odds than males (P < .001), Black race and Hispanic ethnicity with 20% and 26% lesser odds as compared to White (P < .05). Clinical factors included smoking, Alcoholic cirrhosis, Hepatitis B, and Hepatitis C, were statistically significant risk factors of post-liver transplantation malignancies.

**Conclusions:** Malignancies were frequent among elderly patients and predominantly in males. Lymphoproliferative diseases were the most prevalent malignancy types, followed by respiratory/lung cancer- which showed the highest mortality risk of all cancers. LTX patients are at increased risk of head and neck cancers, skin cancers, lymphoma, tumors, and Myelodysplastic syndrome compared to non-LTX patients.

## Introduction

Liver transplantation (LTX) is the second most common organ transplant after Kidney transplantation; 2017 alone witnessed 8,000 liver transplants, and since 2016, 80,000 adults have been living with functional liver grafts 1. Post-liver transplant complications include acute or chronic graft rejection, adverse events associated with immunosuppressive medications, and primary liver disease recurrence. Since long-term immunosuppressive agents are required post-transplantation, their main adverse effect consists of developing malignancies. The literature provides ample evidence that prolonged immunosuppression is associated with carcinogenesis 2-3. Some studies showed that the most frequent *de novo* malignancies (DNMs) in adult LTX recipients are skin, followed by lymphoproliferative diseases 4. Other malignancies include lung cancer, colorectal carcinoma, head and neck cancers 5. Skin cancers are common post-transplant; they are usually localized and respond to local treatment 6, and do not require hospital admission. The risk of developing colorectal cancer is 2 to 3 times higher in LTX recipients, and it increases exponentially in patients getting transplantation secondary to primary biliary sclerosing cholangitis 7.

To the best of our knowledge, few epidemiological studies addressed outcomes in post-liver transplant patients, such as the annual number of hospitalizations, mortality, patient characteristics, and malignancies. This study aimed to determine the nationwide prevalence of malignant neoplasms, and the associated trends, clinical risk factors, patient demographics in hospitalized LTX patients. In addition, we aimed to compare the incidence of malignancies in LTX and non-LTX patients.

## Materials and Methods

### Data Source

We used the National Inpatient Sample (NIS) database of hospitalized patients in the United States with data collected between January 2016 and December 2018, and we only selected data on patients with a primary or secondary diagnosis of liver transplant (LTX) based on an ICD-10 (*International Classification of Diseases, tenth Revision*) code of Z944. All malignant neoplasms were also identified with ICD-10 codes and categorized into Head and Neck, Gastrointestinal (excluding Liver), Bone, Skin, Breast, Reproductive system, Urinary system, Nervous system, Endocrine system, Lymphoma, Leukemia, Myeloma, Tumors, Myelodysplastic Syndrome, Cancer of other sites and Secondary malignancies. The NIS database houses more than 8 million records per year. It is the largest all-payer dataset from the Healthcare Cost and Utilization Project (HCUP), maintained by the Agency of Healthcare Research Quality (AHRQ) 11. NIS upgraded the diagnosis coding from ICD-9 to ICD-10 in September 2015 and redesigned their sampling techniques and weights on participating hospitals' data to optimize accuracy by reducing the margin of error in statistical estimates of outcomes, thus, generating more representation of national estimates. In the database, each patient can have up to 40 diagnoses depending on the dataset year. The dataset is publicly available and lacks patient identification information; thus, Institutional Review Board-IRB approval or informed consent was unnecessary under the Health Insurance Portability and Accountability Act-HIPAA 12.

### Variables and Outcomes

The primary outcome included the prevalence of malignant neoplasms and the corresponding correlation with sociodemographic risk factors in LTX patients. The main variables consisted of patients and hospital characteristics, including age, sex, race, clinical risk factors, socioeconomic status, admission type, admission day, hospital bed size, hospital location, and region. The study population was divided into cases (LTX with malignant neoplasms-except for HCC) and controls (LTX without malignant neoplasms). HCC is considered a common indication for LTX, so we excluded it from the data analysis to avoid bias. The secondary outcomes analyzed the hospital utilization, including discharge status, inpatient mortality, length of stay (LOS), and inpatient hospital-related total cost of care for all malignant neoplasms. The secondary outcome also includes a comparative analysis of the incidence of malignancies among LTX and non-LTX patients.

### Statistical Analysis

The statistical analyses were performed using the SAS statistical software (SAS Institute Inc., Cary, NC, United States). We used mean (±standard deviation-SD) or median (interquartile range-IQR= Q3-Q1) for continuous variables such as age, total charges, and length of stay (LOS). Percentages denoted categorical variables. We performed group comparisons based on sociodemographic characteristics (age, sex, race, median socioeconomic status by national quartile) using Student t-test (for continuous variables) and Chi-square tests (for categorical variables). We used t-tests to calculate the difference in LOS and total charges. Age was categorized into five groups for group-level comparisons (<18; 18-49; 50-59; 60 to 69; and >=70 years of age). A multivariate model developed from the stepwise logistic regression was used to test the predictor variables' association with malignant neoplasms. This regression model selected the most relevant variables to retain at a significant effect level of entry of 0.15 and a level of staying of 0.10, removing other variables not fitting this effect criterion. We calculated odds ratios from logistic regression models, examined covariates' effects on the predictor variables, and adjusted these odds ratios (adjusted aOR) for confounders such as age, gender, and race. We selected a non-LTX matched group based on age, sex, and race using the 1:1 ratio nearest neighbor (greedy) propensity score method. The goodness of fit model was evaluated with Pearson's Chi-square. All hypothesis testing used a two-tailed p-value with the significance level set at 0.05. The missing data on race, primary payer, median household income, discharge status, hospital location, and hospital teaching status were labeled with “other” or “Unknown”.

## Results

A total of 26225 hospital admissions occurred between 2016 and 2018 with liver transplant status, of which 1909 (7.28%) entries were associated with malignant neoplasms (except HCC)- thus constituted our group of cases of hospitalized LTX patients.

### Admission Rate of Malignant Neoplasms

**Figure 1:**
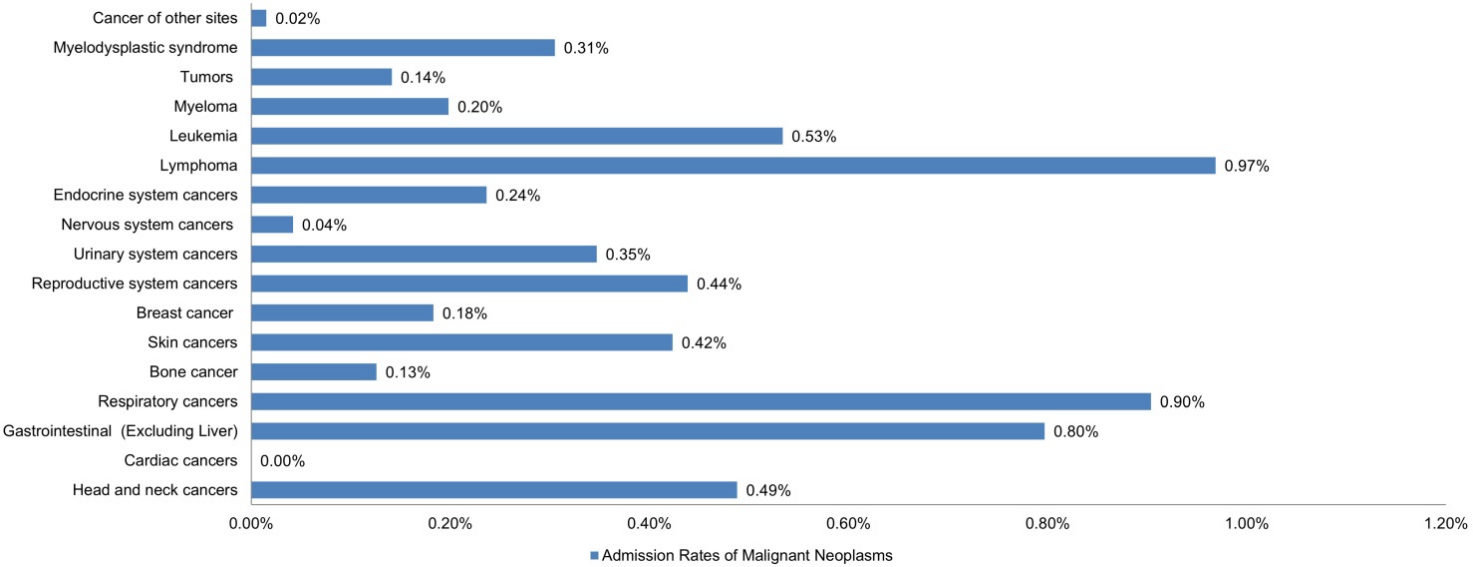
Bar Chart of Admission Rates of Malignant Neoplasms in Liver Transplant Patients.

Lymphomas were the most prevalent cancer among LTX patients, followed by respiratory cancers, gastrointestinal cancers (excluding liver or HCC), leukemia, and head/neck cancers with estimated admission rates of 0.97%, 0.90%, 0.80%, 0.53%, and 0.49%, respectively. Cardiac cancers were the least prevalent, followed by cancers of the nervous system and bone (**Figure 1**).

### Patient Demographics and clinical risk factors

**Figure 2:**
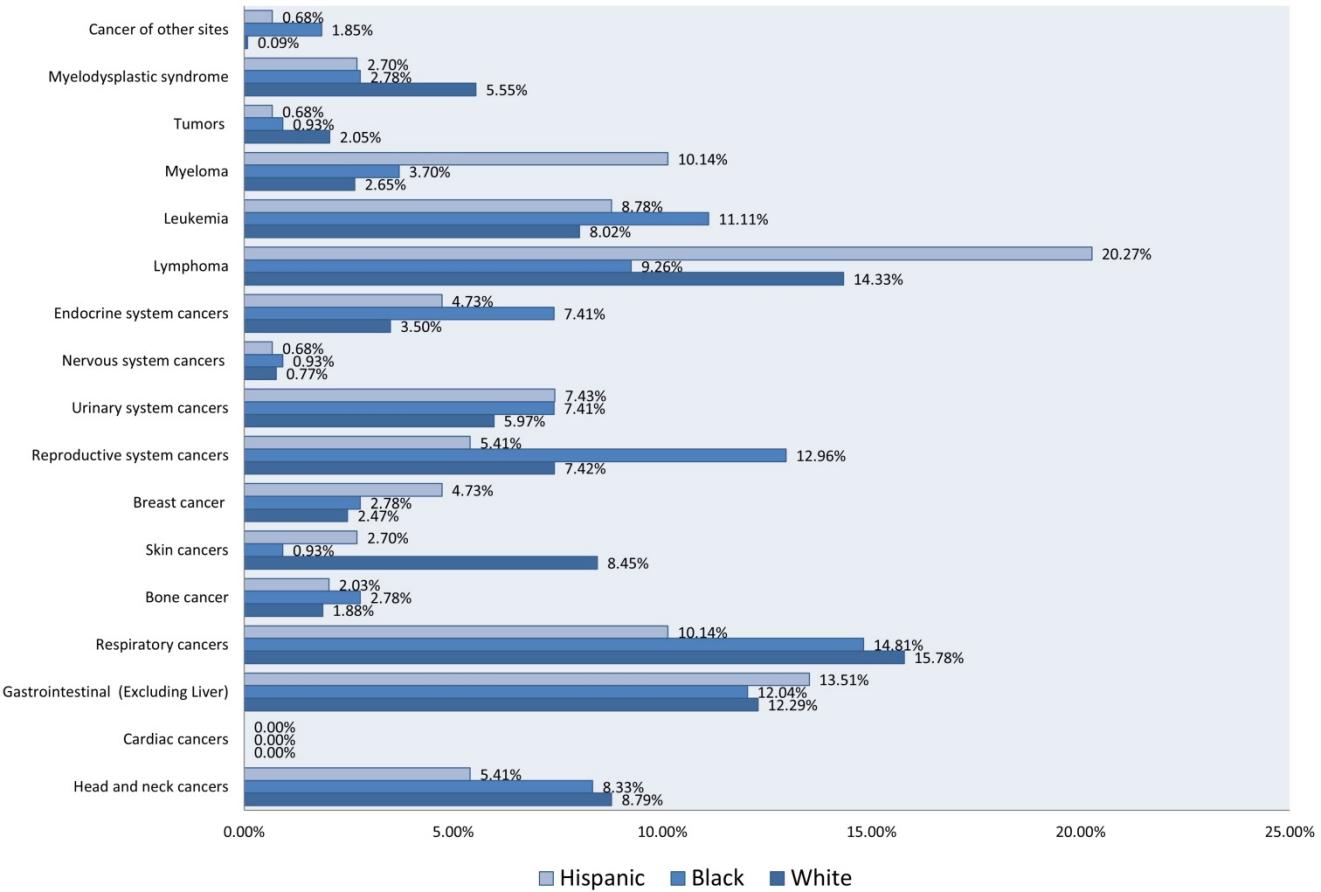
Bar Chart of Rates of Malignant Neoplasms in 1677 Patients, Stratified by Race and Ethnicity. (1356 White, 129 Black, and 192 Hispanic).

Malignant neoplasms with LTX were more frequent in older patients with a mean age of 61.21 years (*SD* ±15.08) and among males (68.73% among cases vs. 59.20% among controls) than females (31.27% for cases vs. 40.80% for controls) (**Table 1**). Respiratory cancer was the most prevalent malignant neoplasm among White patients (15.78%), followed by lymphoma (14.33%) and gastrointestinal cancer (12.29%). Among Black patients, respiratory cancer (14.81%), reproductive system cancer (12.96%), and leukemia (11.11%) were the most reported. Among Hispanics, lymphoma (20.27%) and gastrointestinal cancers (13.51%) prevailed (**Figure 2**).

Malignant neoplasms with LTX were more frequent in patients with smoking (37.56% among cases vs. 32.10% among controls), *p* < .01, alcoholic cirrhosis (3.51% among cases vs. 2.61% among controls) , *p* < .05, hepatitis B (3.09% among cases vs. 1.69% among controls) , *p* < .01, hepatitis C (12.94% among cases vs. 9.83% among controls) , *p* < .01, and opioids (3.72% among cases vs. 2.94% among controls), *p* =0.05. Immunosuppressants, HIV and Primary sclerosing cholangitis (PSC) were also more frequent in patients with LTX but was found to be statistically not significant.

**Table 2** showed the univariate and multivariate regression analyses and some selected sociodemographic and clinical risk factors of malignant neoplasms in hospitalized LTX patients. The multivariate logistic regression showed that female patients were 29% less likely to develop cancers compared to males (*aOR*, 0.712; 95% CI, 0.643 - 0.788; *p* < .001). Compared to patients aged 17 years or less, the odds of cancers in patients aged 18-49 were the same; however, in patients aged 50-59, the odds of cancers were 1.68 times higher (*aOR*, 1.683; 95% CI, 1.294 - 2.190); *p* < .001), 2.1 times higher in patients aged 60-69 (*aOR*, 2.091; 95% CI, 1.625 - 2.690; *p* < .001), and 2.69 times higher in those aged over 70 (*aOR*, 2.685; 95% CI, 2.067 - 3.488; *p* < .001). In terms of race, compared to White patients, Black patients had 20% (*aOR*, 0.802; 95% CI, 0.663 - 0.970; *p* < .05) and Hispanic patients had 24% lesser odds (*aOR*, 0.759; 95% CI, 0.647- 0.889; *p* < .001) of developing malignant neoplasms. Asian or Pacific Islander and Native American odds were not statistically significant. The median household income using national quartiles ranging from 1 (lowest median income) to 4 (highest median income) was utilized in the regression model as a proxy measure reflecting patients' socioeconomic status based on their communities' zip codes. Compared to median household income in quartile 1, the odds of cancers in patients with median household income in quartile 3 were 1.28 times (*aOR*, 1.285; 95% CI, 1.121 - 1.473; *p* < .001), and 1.16 times (*aOR,* 1.161; 95% CI, 1.004 - 1.342; *p* <.05) in quartile 4.

LTX Patients have 12% greater odds to develop cancers with smoking (*aOR*, 1.121; 95% CI, 1.013 - 1.240; *p* < .05), 30% greater odds with alcoholic cirrhosis (*aOR*, 1.1298; 95% CI, 1.0 - 1.687; *p* = .051), 54% greater odds with hepatitis B (*aOR*, 1.554; 95% CI, 1.166 - 2.071; *p* < .01), 24% greater odds with hepatitis C (*aOR*, 1.237; 95% CI, 1.069- 1.431; *p* < .01), and 36% greater odds with opiods (*aOR*, 1.362; 95% CI, 1.056 - 1.756; *p* < .01).

Patients with malignant neoplasms had 73% greater odds of elective admissions than non-elective admissions (*aOR*, 1.731; 95% CI, 1.540 - 1.946; *p* < .001), and 27% more likely to get admitted to medium hospitals as compared to small (*aOR*, 1.277; 95% CI, 1.065 - 1.532; *p* < .005), and higher likelihood to get admitted into large hospitals (47%) than to small (*aOR*, 1.473; 95% CI, 1.253 - 1.732; *p* < .005). In our study, the hospital's teaching status and its region failed to show a statistically significant association with risk for malignant neoplasm. Regarding discharge characteristics, cases were 34% more likely to transfer to home health care (HHC) as compared to routine discharges (*aOR*, 1.342; 95% CI, 1.190 - 1.514; *p* < .001), and had 3.9 times greater odds of in-hospital mortality (*aOR*, 3.293; 95% CI, 2.616 - 4.145; *p* < .001). The mean differences in total length of hospital stay (LOS) and hospital inpatient charges were found to be higher in LTX patients with malignant neoplasms (0.89; 95% CI, 0.52 - 1.26; *p* < .001) and ($12132 95% CI, $6757 - $17508; *p* < .001), respectively.

### Incidence of Malignant Neoplasms in LTX patients and matched Cohort of Non-LTX patients

Table 3 showed the incidence of Malignant Neoplasms in LTX patients and a matched cohort of non-LTX patients. We selected a matched cohort utilizing propensity score method based on patients age, sex, and race. The comparison showed that LTX patients are at increased risk of head and neck cancers, skin cancers, lymphoma's, tumors, and Myelodysplastic syndrome. The incidence of head and neck cancer after liver transplantation is 1.5 times higher (0.49% among LTX vs. 0.33% non-LTX; *p* < .01), 3.2 times higher in skin cancer (0.49% among LTX vs. 0.33% non-LTX; *p* < .01), 1.4 times higher in lymphoma's (0.97% among LTX vs. 0.69% non-LTX; *p* < .01), 1.58 times higher in Myelodysplastic syndrome (0.30% among LTX vs. 0.19% non-LTX; *p* < .01), and 1.55 times higher in tumors (0.14% among LTX vs. 0.09% non-LTX; *p* = .096).

### Hospital Utilization and Discharge Characteristics

**Figure 3:**
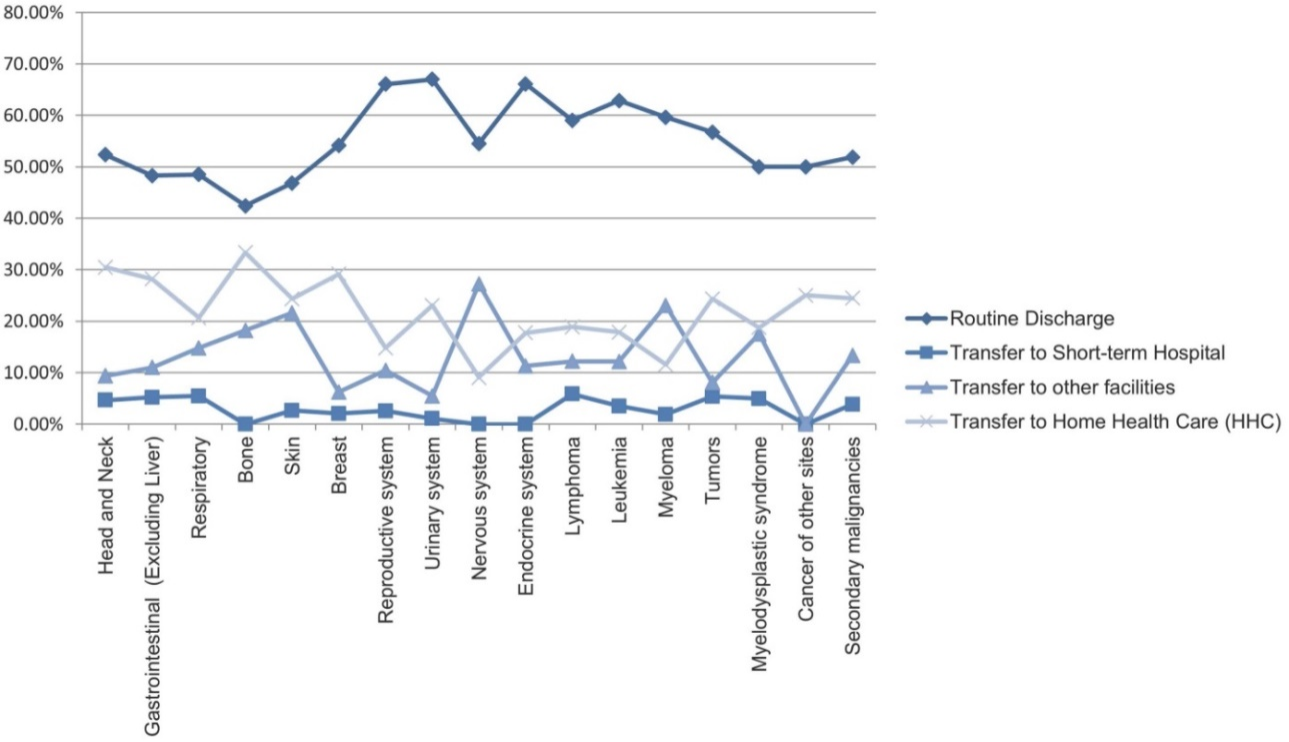
Bar Plots of Length of Stay (mean and standard deviation) of all Malignant Neoplasms in LTX Patients.

**Figure 4:**
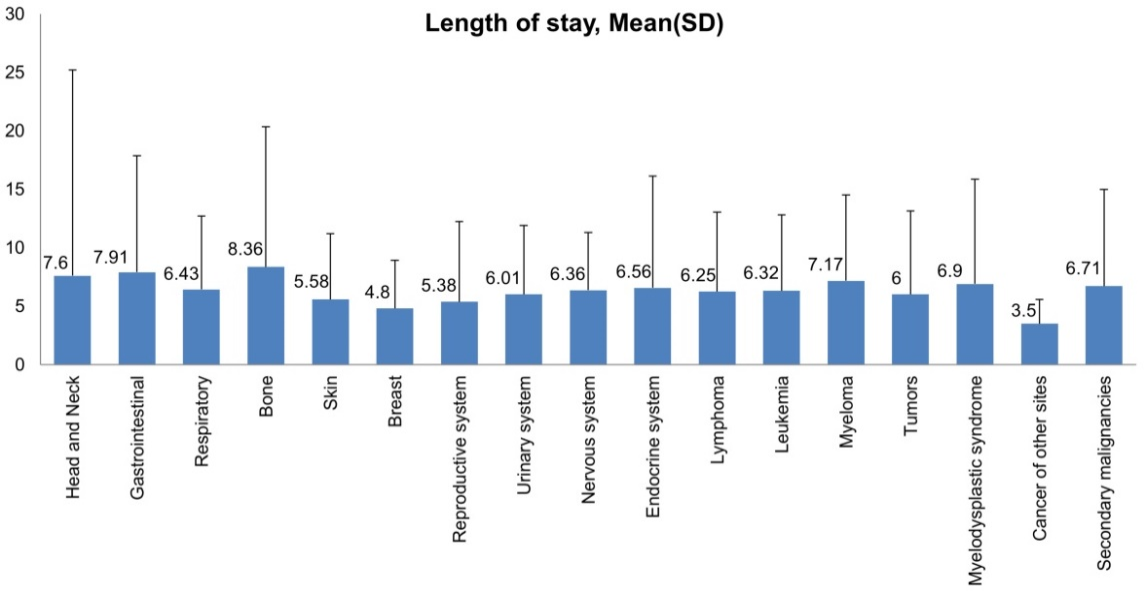
Bar Plots of Mean Total Charges of all Malignant Neoplasms in LTX Patients.

**Figure 5:**
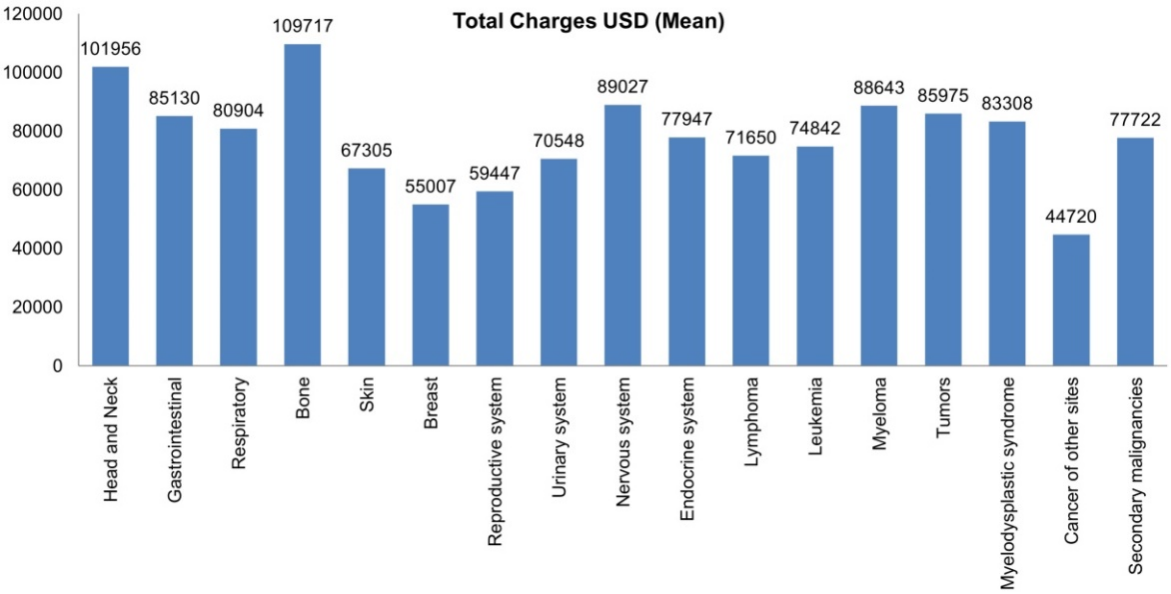
Bar Plots of in-hospital Mortality all Malignant Neoplasms in LTX Patients.

**Figure 6:**
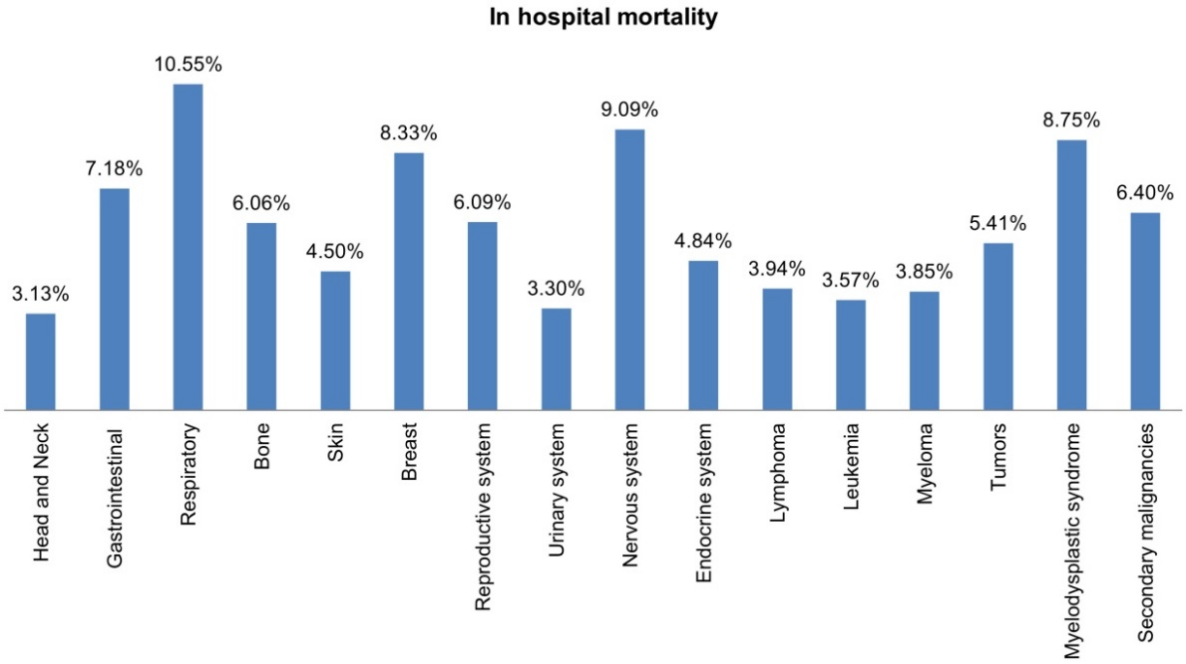
Line Graph of Discharge Rate (Proportions) of all Malignant Neoplasms in LTX Patients.

Most hospital admissions were non-elective (78.24% among cases vs. 86.16% among controls) as compared to elective (21.76% among cases and 13.84% among controls), *p* < .001, more patients admitted on weekdays- 80% compared to around 20% during weekdays and weekends, respectively in both cases and controls (*p* < .001). In both groups, more than 65% of patients got admitted to large hospitals (68.88% of cases vs. 65.10% of controls) compared to 21% to medium hospitals and 10% to small hospitals (*p* < .001). In terms of hospital teaching status, most patients with LTX preferred admission to an urban teaching hospital (84.60% for cases and 84.40% for controls) than urban nonteaching and rural hospitals (**Table 1**). Patients with LTX and malignant neoplasms (cases) had a higher length of stay (LOS) compared to LTX patients (controls), *M* = 6.45 days (*SD* ±8.54) vs. *M* = 5.56 days (*SD* ±7.94), respectively (**Table 4**). Similarly, **Table 4** showed that cases had a higher inpatient hospital cost of care than controls with a respective mean total charge of $77,127 (*SD* ±$102,652) vs. $64994 (*SD* ±$115,960). Patients admitted with bone, gastrointestinal, head, neck, and myeloma cancers had a higher mean length of stay (LOS) than other cancers (**Figure 3).** In terms of total charges, malignant bone neoplasm had the highest total charges, followed by head and neck cancers, nervous system cancers, and myeloma (**Figure 4).** Respiratory neoplasm had the highest in-hospital mortality followed by cancer of the nervous system, myelodysplastic syndrome, and breast cancers with a mortality rate of 10.55%, 9.09%, 8.75%, and 8.33%, respectively **(Figure 5)**. For discharge status, all cases had the highest routine discharges and lowest discharges to short-term hospitals. Furthermore, most cases witnessed a higher transfer rate to home healthcare than transfer to other facilities (including Skilled Nursing Facility and Intermediate Care Facility) - except for nervous system cancers and myeloma** (Figure 6)**.

## Discussion

Previous studies demonstrated that liver transplant recipients were at risk of developing *de novo* malignancies 2, 3, 4, 5, 6, 7, 8, 13, 14, 23, 24, 26. Fewer studies reported a 3-7 times greater risk for malignancy among LTX recipients than the general population 13. Based on the data from the literature, the incidence rate of malignancies after liver transplant ranged from 4% to 16%. Our study found a total of 26225 hospital admissions with liver transplant status occurred in the three years from 2016 to 2018, of which 1909 admissions (7.28%) had malignant neoplasms excluding hepatocellular carcinoma. The most common reported malignancy among admitted patients was lymphoma, followed by respiratory cancers and gastrointestinal cancers. Based on the literature, the hazard of developing malignancy is one out of every six patients within 20 years of liver transplantation 14. Previous studies discussed multiple risk factors related to the occurrence of malignancies in post-transplant patients. They included the following: recipient age, excess immunosuppression, alcohol liver disease, smoking, hepatitis B, hepatitis C, HIV, Kaposi sarcoma, and EBV seropositivity of donor and recipient 13, 28. In our study, smoking, Alcoholic cirrhosis, Hepatitis B, and Hepatitis C, were statistically significant risk factors of post-liver transplantation malignancies. Although, more opioid use was seen among the malignant cohort. Among virus-related malignancies, non-Hodgkin lymphomas were the most reported neoplasms in liver transplant patients 3. In our study, LTX patients with long-term immunosuppressive agents were at a higher risk of malignant neoplasm (0.84% among cases vs. 0.75% among control). However, they were not statistically significant, possibly due to fewer patients and limitations of the NIS dataset. In addition, we could not compare necessary information like the blood concentration, dose, and the duration of the immunosuppressants. Immunosuppressive therapy in organ transplant recipients predisposes them to cancer by downplaying the immune response against the malignant antigens. Immunosuppressant has dose-dependent effects; the higher the drug dose, the higher the risk of *de novo* malignancy or recurrence of hepatocellular carcinoma 15, 22. Few *in-vitro* studies 16 showed that anti-rejection drugs could have pro-oncogenic activity by overexpressing the transforming growth factor-beta (TGF-ß), causing impairment of signaling pathways and dysregulation of the immune system, thus, promoting the development and progression of aggressive malignancy 27. Hence, similarly to the dose-effect, the longer the duration of immunosuppressive agents, the higher the risk for malignancies 17. Viral infections constitute another critical risk factor for neoplasm development. Viruses modulate and disrupt the normal cell-cycle functions of the host cells; their encoded viral genes sabotage the immune system, thus, increase the risk of tumorigenesis 18. In the United States, the incidence of malignancy transmission from deceased donors is <1% 19.

Our study showed that the risk of developing malignant neoplasms in LTX patients increases with age. Also, malignant neoplasms were more common in males than females (29% lesser odds- aOR, 0.712; 95% CI, 0.643 - 0.788; p < .001). Black and Hispanic patients were at lower risk than Whites. Our study also showed that smoking is a significant risk factor associated with higher odds of malignant neoplasms in LTX patients. 12% greater odds to develop cancers with smoking (aOR, 1.121; 95% CI, 1.013 - 1.240; p < .05. LTX patients had 36% greater odds of opioids use (aOR, 1.362; 95% CI, 1.056 - 1.756; p < .01).

### Post-transplant Lymphoproliferative Disease

Our study revealed that the most common malignancies present among hospitalized LTX patients are lymphomas, followed by respiratory cancers. Gastrointestinal cancers, excluding hepatocellular carcinoma, marked the third top malignancy. In our population dataset, lymphomas accounted for 0.97% of admissions in post-liver transplant patients, with the highest incidence among Hispanics (20.27%). lymphomas or lymphoproliferative disorders in the post-transplant phase could correlate with inadequate immune response toward the EBV virus 15. Other responsible viruses are hepatitis B and hepatitis C for liver cancer and HPV in squamous cell carcinoma 28. One of the protective factors for lymphoma and leukemia is to use alternative immunosuppressive agents like mycophenolate mofetil 20.

### Respiratory Cancers

Respiratory malignant neoplasms are the second leading in our LTX patient's cohort. Often lung cancer is seen in lung transplant patients or heart transplant recipients 21. The incidence of lung or bronchus cancer in organ transplant recipients is higher among smokers- as expected. Some studies have suggested a potential association between increased risk for respiratory cancers and the use of immunosuppression with anti-lymphocyte globulins for induction therapy and rejection prevention after transplantation 22. Based on our results, 0.90% of LTX patients developed respiratory/lung cancer, which was the most common neoplasm in Whites, accounting for 15.78%.

### Gastrointestinal Malignancies

Gastrointestinal cancers, excluding hepatocellular carcinomas, ranked third as the most common malignancies in our study population. Out of all the GI malignancies, colorectal malignancies in LTX patients were the most studied. Previous studies showed that the incidence rate of colorectal cancer is 0.4 to 0.54 % in the United States 23. Our study showed that 0.8% of LTX patients' admissions were related to GI malignancies (excluding HCC). It is the second most common cancer among Hispanics (13.51%). The literature revealed that patients with primary sclerosing cholangitis (PSC) associated with inflammatory bowel disease (IBD) are at high risk for developing GI malignancies 26. The exact pathogenesis for increasing the risk of colorectal cancer with PSC is not known. One hypothesis is that inflammation can expedite the process. Patients with PSC and IBD require more aggressive immunosuppression, thus inadvertently risking any *de novo* malignancy 24.

### Other Malignancies

Other cancers observed in LTX patients included head and neck, genitourinary, nervous system, bone, skin, leukemia, myeloma, breast, and endocrine cancers. Data on admissions with cardiac cancers were missing. Head, neck, and bone cancers had lesser admission rates (0.49% and 0.13%), but their inpatient hospital charges were higher, and their length of stay was more extended than more prevalent cancers like lymphomas.

### Comparative analysis of Incidence of Malignant Neoplasms in LTX patients and matched Cohort of Non-LTX patients

Our study revealed that LTX patients are at increased risk of head and neck cancers, skin cancers, lymphoma, tumors, and Myelodysplastic syndrome compared to our non-LTX hospitalized patient cohort. The highest on the list is skin cancer with 3.2 times higher odds, which aligns with the previous studies' results 25, followed by myelodysplastic syndrome with 1.58 times higher odds. Head and Neck cancers and lymphomas are third and fourth on the list.

### Hospital Burden and Mortality

This study highlighted that most admissions of LTX patients with malignancy were elective and were on weekdays. Patients preferred sizeable urban teaching hospitals over small urban non-teaching hospitals. LTX patients with malignant neoplasms had a longer length of stay with a mean of 6.45 days as compared to 5.56 days of LTX patients with no malignancies. This longer length of stay can be attributed to more comorbidities and complications in this patient population. Our results showed that bone cancers had the most extended length of stay, possibly because of widespread metastasis and malignancy complications. In-hospital mortality was 3.3 times higher in LTX patients with malignant neoplasms than in those without malignancy. Respiratory cancers accounted for the highest inpatient mortality. Inpatient hospital cost of care was higher in LTX patients with malignant neoplasms secondary to more complicated hospital courses and expensive chemotherapy treatment regimens- thus increasing the health care burden. Out of all malignancies, bone cancers recorded the highest utilization of hospital charges. This finding is in line with our previously mentioned observation of bone cancer patients' most extended length of stay.

### Limitations and strengths

We used the National Inpatient Sample database with data collected between January 2016 and December 2018. With more than 7 million records per year, the NIS administrative database could be prone to selection bias and coding errors. Our study encountered multiple limitations; for instance, liver donor information was lacking from the database, and one of the significant risk factors of malignancy in post LTX patients includes EBV seropositivity in the donor.Another limitation was the missing data regarding the length of time (the interval duration) between transplantation and the incidence of diagnosis of malignancy. In addition, the duration and dosage of immunosuppressant drugs were not available. Moreover, since this database housed inpatients data only, we could not evaluate any outpatient follow-up and previous cancer screening. Therefore, the calculated mortality from malignant neoplasms reflects only that for the inpatient sample and does not account for outpatient mortality rates; therefore, it could be that our data present an underestimation of malignancy-associated mortality rates post liver transplant.

This study remains unique because it highlights the impact of malignancy post-liver transplant using a large and diverse cohort of patients with national data from all United States regions compared to previous studies of specific geographical areas or centers. In addition, we compared the LTX patients with a non-LTX matched cohort and compared the incidence of various cancers. To the best of our knowledge, it is the first study from the National Inpatient Sample database to analyze hospital utilization and the discharge status of all liver transplant patients. Despite the inherent pitfalls of using an administrative database to study clinical outcomes, still, the NIS database houses a plethora of national data that provide a rough estimation of the cumulative prevalence of malignancy post-liver transplant at the national level; thus, its utilization can be harnessed to generate new hypotheses for prospective controlled experimental studies.

## Conclusion

The number of post-liver transplantation complications is on the rise. Therefore, our study aimed to observe the incidence of malignancies in hospitalized liver transplant patients. Among these hospitalized LTX patients, lymphoproliferative diseases followed by respiratory cancers were the most reported malignancies. Moreover, our research found that LTX hospitalized patients are at increased risk of head and neck cancers, skin cancers, lymphoma, tumors, and Myelodysplastic syndrome than non-LTX patients.

This study highlights a clear trend in the patient sociodemographic and discharges characteristics. Advanced age, being White, and being male were significantly associated with a higher likelihood of malignant neoplasms in liver transplant patients. Patients aged 70 and up were 2.7 times more likely to develop malignancy than young patients; females had 29 % lesser odds of malignancy than males.

In our research, factors like smoking, Alcoholic cirrhosis, Hepatitis B, Hepatitis C were statistically significant risk factors of post-liver transplantation malignancies. Moreover, our study also found opioids linked with a higher rate of post-liver transplantation malignancies.

Respiratory cancer had the highest risk of mortality among all cancers, accounted for 10.5% of all reported mortalities. Cancers of the nervous system, myelodysplastic syndrome, and breast cancer admission rates were lower, but their mortality was higher than other cancers with 9.1%, 8.75%, and 8.3%. Although head, neck, and bone cancers were uncommon in LTX patients, they had a longer length of stay and hospital charges than more frequent cancers like lymphomas and respiratory. Most of the patients had routine discharge, followed by transfer to home health care, other facilities (Includes Skilled Nursing Facility, Intermediate Care Facility), or short-term hospitals.

To conclude, regular cancer screening in liver transplant patients is crucial as early detection helps in enhancing treatment responsiveness. Therefore, clinicians should proactively seek to diagnose these aggressive cancers post-liver transplantation as early as possible to reduce morbidity and mortality, hospital burden, and improve patients' outcomes and quality of life.

## Author Contributions

The authors confirm contribution to the paper as follows:

Study conception and design: Author, Maryam Bilal Haider (corresponding author). Data collection and statistical analysis: Author, Syed M Haider. Interpretation of results: Author, Rana Ismail. Draft manuscript preparation: Author, Maryam Bilal Haider, and Anusha Bapatla. Agreement to be accountable for all aspects of the work in ensuring that questions related to the accuracy or integrity of any part of the work are appropriately investigated and resolved: Author, Rana Ismail, Ahmed J Chaudhary, and Sana Iqbal.

All authors reviewed the results and approved the final version of the manuscript.

## Figures and Tables

**Table 1:** Baseline Patient Demographics with Liver Transplant, with and without Malignant Neoplasms.

Variables	Malignant Neoplasms		*P* value
	No (n= 24316) 92.72%	Yes (n= 1909) 7.28%	
Sex			<0.01^2^
Female	9922 (40.80%)	597 (31.27%)	
Male	14394 (59.20%)	1312 (68.73%)	
Age (y), mean (SD)	55.23 (18.99)	61.21 (15.08)	<0.01^1^
Age groups (y)			
<=17	1829 (7.52%)	72 (3.77%)	
18-49	4236 (17.42%)	165 (8.64%)	
50-59	4984 (20.50%)	359 (18.81%)	
60-69	8923 (36.70%)	814 (42.64%)	
>=70	4344 (17.86%)	499 (26.14%)	
Race/Ethnicity			<0.01^3^
White	15885 (65.33%)	1356 (71.03%)	
Black	2281 (9.38%)	129 (6.76%)	
Hispanic	3366 (13.84%)	192 (10.06%)	
Asian or Pacific Islander	652 (2.68%)	67 (3.51%)	
Native American	182 (0.75%)	9 (0.47%)	
Other	1950 (8.02%)	156 (8.17%)	
Median socioeconomic status by national quartiles			<0.01^3^
0-25	6132 (25.22%)	401 (21.01%)	
25-50	6158 (25.32%)	475 (24.88%)	
50-75	6300 (25.91%)	565 (29.60%)	
75-100	5303 (21.81%)	447 (23.42%)	
Other	423 (1.74%)	21 (1.10%)	
Admission Type			<0.01^2^
Non-elective	20921 (86.16%)	1492 (78.24%)	
Elective	3361 (13.84%)	417 (21.76%)	
Admission Day			<0.01^2^
Weekdays	19421 (79.87%)	1574 (82.45%)	
Weekend	4895 (20.13%)	335 (17.55%)	
Hospital Bed Size			<0.01^3^
Small	2961 (12.18%)	185 (9.69%)	
Medium	5525 (22.72%)	409 (21.42%)	
Large	15830 (65.10%)	1315 (68.88%)	
Location/teaching status of hospital			NS
Rural	1030 (4.24%)	76 (3.98%)	
Urban nonteaching	3250 (13.37%)	218 (11.42%)	
Urban teaching	20036 (84.40%)	1615 (84.60%)	
Hospital region			NS
Northeast	4505 (18.53%)	414 (21.69%)	
Midwest or North Central	5558 (22.86%)	441 (23.10%)	
South	9318 (38.32%)	679 (35.57%)	
West	4935 (20.30%)	375 (19.64%)	
Discharge Characteristics			<0.01^3^
Routine Discharge	15714 (64.92%)	1072 (56.16%)	
Transfer to Short-term Hospital	1093 (4.49%)	76 (3.98%)	
Transfer to other facilities	3108 (12.78%)	234 (12.26%)	
Home Health Care (HHC)	4008 (16.48%)	423 (22.16%)	
In hospital mortality	393 (1.62%)	104 (5.45%)	
Length of stay (days), mean (SD)	5.5617 (7.94)	6.4547 (8.54)	<0.01^1^
Total charges (USD), mean (SD)	64994 (115960)	77127 (102652)	<0.01^1^
Malignancies Risk Factors			
Smoking	7806 (32.10%)	717 (37.56%)	<0.01^2^
Alcoholic cirrhosis	634 (2.61%)	67 (3.51%)	0.0185
Hepatitis B	410 (1.69%)	59 (3.09%)	<0.01^2^
Hepatitis C	2390 (9.83%)	247 (12.94%)	<0.01^2^
HIV	120 (0.49%)	12 (0.63%)	NS
Immunosuppressants	183 (0.75%)	16 (0.84%)	NS
Opioid Use	715 (2.94%)	71 (3.72%)	0.05
Primary sclerosing cholangitis	56 (0.23%)	6 (0.31%)	NS

^1^ Two sample Student *t*-test, 2-tailed for comparing means of two Continuous Variables. ^2^ Pearson Chi-Square 2-tailed Test for association of two Categorical Variables. ^3^Pearson Chi-square, 2-tailed Test for 2 by *n* table. Statistical significance illustrates that two group differs. NS: Not statistically significant.

**Table 2:** Risk Factors of Malignant Neoplasms in Hospitalized Patients with Liver Transplant Status in the United States- from 2016 to 2018.

Malignant Neoplasms	Odds Ratio (95%CI)	*P* value	Adjusted Odds Ratio (95%CI)	*P* value
	Univariate logistic regression^1^		Multivariate logistic regression^2^	
Sex, Female vs Male	0.660 (0.597 - 0.730)	<.001	0.712 (0.643 - 0.788)	<.001
Age groups (y)				
<=17	Reference	NA	Reference	NA
18-49	0.989 (0.746 - 1.312)	NS	0.976 (0.734 - 1.299)	NS
50-59	1.830 (1.413 - 2.370)	<.001	1.683 (1.294 - 2.190)	<.001
60-69	2.317 (1.812 - 2.964)	<.001	2.091 (1.625 - 2.690)	<.001
>=70	2.918 (2.265 - 3.758)	<.001	2.685 (2.067 - 3.488)	<.001
Race/ethnicity				<.001
White	Reference	NA	Reference	NA
Black	0.663 (0.550 - 0.798)	<.001	0.802 (0.663 - 0.970)	<.05
Hispanic	0.668 (0.572 - 0.781)	<.001	0.759 (0.647- 0.889)	<.001
Asian or pacific islander	1.204 (0.931 - 1.557)	NS	1.219 (0.938 - 1.585)	NS
Native American	0.937 (0.789 - 1.113)	NS	0.789 (0.401 - 1.552)	NS
Median socioeconomic status by national quartiles				
0-25	Reference	NA	Reference	NA
25-50	1.180 (1.028 - 1.353)	NS	1.117 (0.971 - 1.285)	NS
50-75	1.371 (1.201 - 1.566)	<.001	1.285 (1.121 - 1.473)	<.001
75-100	1.289 (1.121 - 1.482)	NS	1.161 (1.004 - 1.342)	<.05
Admission Type, Elective vs Non-elective	1.731 (1.544 - 1.942)	<.001	1.731 (1.540 - 1.946)	<.001
Hospital Bed Size				
Small	Reference	NA	Reference	NA
Medium	1.185 (0.990 - 1.417)	NS	1.277 (1.065 - 1.532)	<.005
Large	1.329 (1.134 - 1.558)	<.001	1.473 (1.253 - 1.732)	<.001
Location/teaching status of hospital				
Rural	Reference	NA	Reference	NA
Urban nonteaching	0.909 (0.694 - 1.191)	NS	0.883 (0.669 - 1.164)	NS
Urban teaching	1.092 (0.861- 1.387)	NS	1.162 (0.908 - 1.487)	NS
Hospital region				
Northeast	Reference	NA	Reference	NA
Midwest or North Central	0.863 (0.751 - 0.993)	NS	NA	NS
South	0.793 (0.698 - 0.901)	<.001	NA	NS
West	0.827 (0.715 - 0.956)	NS	NA	NS
Discharge Characteristics				
Routine Discharge	Reference	NA	Reference	NA
Transfer to Short-term Hospital	1.019 (0.801 - 1.297)	NS	1.093 (0.856 - 1.397)	NS
Transfer to other facilities	1.104 (0.953 - 1.278)	NS	0.951 (0.818 - 1.106)	NS
Home Health Care (HHC)	1.547 (1.375 - 1.740)	<.001	1.342 (1.190 - 1.514)	<.001
Smoking	1.273 (1.156 - 1.402)	<.001	1.121 (1.013 - 1.240)	<.05
Alcoholic cirrhosis	1.360 (1.053 - 1.757)	0.0186	1.298 (1.0 - 1.687)	0.0507
Hepatitis B	1.860 (1.410 - 2.454)	<.001	1.554 (1.166 - 2.071)	<.01
Hepatitis C	1.365 (1.186 - 1.570)	<.001	1.237 (1.069 - 1.431)	<.01
HIV	1.276 (0.704 - 2.314)	NS	NA	NS
Immunosuppression	1.115 (0.667 - 1.863)	NS	NA	NS
Opioid	1.276 (0.995 - 1.636)	0.0546	1.362 (1.056 - 1.756)	<.05
PSC	1.367 (0.589 - 3.177)	NS	NA	NS

^1^Univariate logistic regression is performed in SAS software with PROC Logistic. ^2^Mutivariate logistic regression is performed with stepwise logistic regression with a 0.15 significance level of entry and 0.10 significance level of stay. NS: Not statistically significant.

**Table 3:** Comparison of inpatient mortality, mean total charges and length of stay between liver transplant patients with malignant neoplasms and liver transplant patients without malignant neoplasms, 2016-2018.

	Mortality		Length of Stay		Total Charges	
	Adjusted Odds Ratio (95%CI)	*P* value	Difference (95%CI)	*P* value	Difference (95%CI)	*P* value
LTX with Malignant Neoplasms	3.293 (2.616 - 4.145)	<.001	0.8930 (0.5210 - 1.2650)	<.001	$12132.8 ($6757 - $17508)	<.001
LTX without Malignant Neoplasms	Reference	NA	Reference	NA	Reference	NA

**Table 4:** Incidence of Malignant Neoplasms in LTX patients with Vs. Matched cohort of non-LTX patients.

Variables	1:1 matched comparison of LTX & non-LTX patients		*P* value
	LTX = No (n= 26225) 50%	LTX = Yes (n= 26225) 50%	
Head and neck	87 (0.33%)	128 (0.49%)	0.005
Cardiac	3 (0.01%)	0 (0.00%)	NS
Gastrointestinal	375 (1.43%)	209 (0.80%)	<.0001
Respiratory	338 (1.29%)	237 (0.90%)	<.0001
Bone	48 (0.18%)	33 (0.13%)	0.0953
Skin	33 (0.13%)	111 (0.42%)	<.0001
Breast	95 (0.36%)	48 (0.18%)	<.0001
Reproductive	268 (1.02%)	115 (0.44%)	<.0001
Urinary	140 (0.53%)	91 (0.35%)	0.0012
Nervous system	70 (0.27%)	11 (0.04%)	<.0001
Endocrine	98 (0.37%)	62 (0.24%)	0.0044
Lymphoma	182 (0.69%)	254 (0.97%)	0.0005
Leukemia	204 (0.78%)	140 (0.53%)	0.0005
Myeloma	86 (0.33%)	52 (0.20%)	0.0038
Tumors	24 (0.09%)	37 (0.14%)	0.0958
Myelodysplastic syndrome	50 (0.19%)	80 (0.30%)	0.0084
Cancers of other sites	6 (0.02%)	4 (0.02%)	NS
Secondary malignancies	764 (2.91%)	719 (2.74%)	0.2359

^1^ Pearson Chi-Square 2-tailed Test for association of two Categorical Variables. NS: Not statistically significant.
